# Roles of Anthrax Toxin Receptor 2 in Anthrax Toxin Membrane Insertion and Pore Formation

**DOI:** 10.3390/toxins8020034

**Published:** 2016-01-22

**Authors:** Jianjun Sun, Pedro Jacquez

**Affiliations:** Department of Biological Sciences, Border Biomedical Research Center, University of Texas at El Paso, 500 West University Avenue, El Paso, TX 79968, USA; prjacquez@utep.edu

**Keywords:** anthrax, ANTXR2, pore formation, membrane translocation, pathogenesis

## Abstract

Interaction between bacterial toxins and cellular surface receptors is an important component of the host-pathogen interaction. Anthrax toxin protective antigen (PA) binds to the cell surface receptor, enters the cell through receptor-mediated endocytosis, and forms a pore on the endosomal membrane that translocates toxin enzymes into the cytosol of the host cell. As the major receptor for anthrax toxin *in vivo*, anthrax toxin receptor 2 (ANTXR2) plays an essential role in anthrax toxin action by providing the toxin with a high-affinity binding anchor on the cell membrane and a path of entry into the host cell. ANTXR2 also acts as a molecular clamp by shifting the pH threshold of PA pore formation to a more acidic pH range, which prevents premature pore formation at neutral pH before the toxin reaches the designated intracellular location. Most recent studies have suggested that the disulfide bond in the immunoglobulin (Ig)-like domain of ANTXR2 plays an essential role in anthrax toxin action. Here we will review the roles of ANTXR2 in anthrax toxin action, with an emphasis on newly updated knowledge.

## 1. Introduction

Tumor endothelial marker 8 (TEM8) was first reported as a gene that is expressed in human tumor endothelium [1], and capillary morphogenesis gene 2 (CMG2) was found to be differentially expressed during capillary morphogenesis in three-dimensional (3D) collagen matrices [2]. Both TEM8 and CMG2 are ubiquitously expressed type I transmembrane proteins and share ~40% overall sequence homology. While little was known about their physiological roles, they have become two of the most intensively investigated molecules since they were identified as anthrax toxin receptors. *Bacillus anthracis*, the causative agent of anthrax, was ever used as a biological weapon by Japanese army during World War II. The 2001 anthrax attack in the US generated great terror in the public and boosted anthrax-related research. A few months later, after 2001 anthrax attack, tumor endothelial marker 8 (TEM8) was reported as an anthrax toxin receptor [3], hence being named anthrax toxin receptor 1 (ANTXR1), which was followed by the identification of CMG2 as the second anthrax toxin receptor (ANTXR2) [4]. Over the past 14 years, intensive research efforts have been spent to investigate the two receptors and a great deal of knowledge has been generated. Anthrax toxin and anthrax toxin receptors have been comprehensively reviewed in several excellent review articles [5,6,7,8,9,10,11]. Here, we provide an updated review of the most recent research progress concerning ANTXR2, the major receptor for anthrax toxin *in vivo*, in anthrax toxin action.

## 2. Anthrax and Anthrax Toxin

To defend themselves against the immune system of mammalian hosts, pathogenic bacteria usually deliver toxins into the cytosol of host cells to disrupt key steps of cellular metabolism and signal transduction. Bacterial A-B toxins or binary toxins are the class of molecules that are perfectly designed by nature to achieve this goal. The B (binding) moiety binds to the cell surface and forms a pore on the membrane. The A (enzymatically active) moiety is usually translocated across the membrane through the pore into the cytosol, where the A moiety executes its catalytic activity, resulting in the disruption of cellular physiological functions [12].

**Figure 1:**
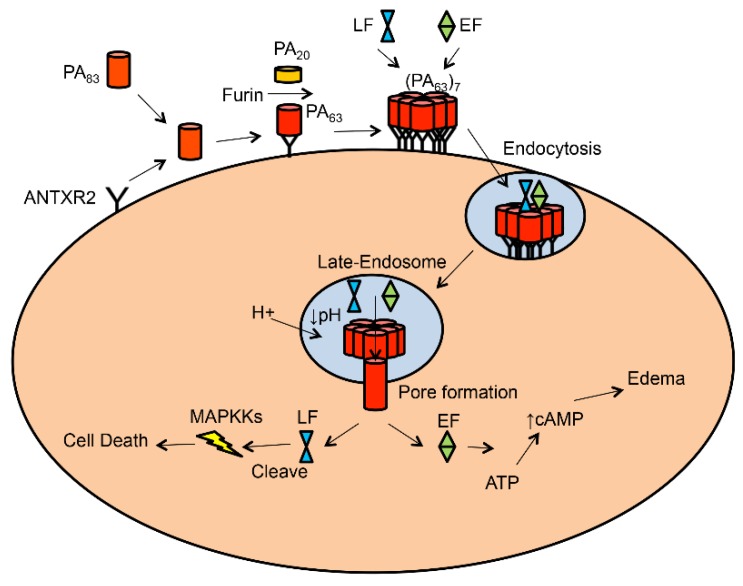
Model of anthrax toxin–mediated intoxication of host cell**.** PA_83_ binds to cell surface receptor and is cleaved by furin into PA_63_ and PA_20_. PA_63_ assembles into a heptameric prepore (PA_63_)_7_, to which LF/EF binds. The complex is internalized by the receptor-mediated endocytosis and travels to the late endosome, where acidification triggers conversion of PA prepore to pore. EF/LF is translocated into the cytosol through the PA pore. In the cytosol, LF cleaves MAPKK and EF elevates cAMP.

Anthrax toxin is a tripartite A-B toxin. It is composed of two catalytic A moieties and one receptor-binding/pore-forming B moiety. The A moieties are edema factor (EF), an 89-kDa calmodulin-dependent adenylate cyclase, and lethal factor (LF), a 90-kDa zinc protease. The B moiety is protective antigen (PA). PA is produced and secreted as a 83-kDa protein [6]. PA_83_ binds to the cell surface receptor and is cleaved by cellular furin or a furin-like protease to generate an active, 63-kDa form (PA_63_) [13]. PA_63_ is self-assembled into a heptameric or octameric receptor-bound prepore, which contains high-affinity binding sites for EF and LF [14,15]. The toxin-receptor complexes are then engulfed into the cell through a receptor-mediated, clathrin-dependent endocytosis, which requires post-translational modifications (e.g., ubiquitination and phosphorylation) on the receptor cytosolic domain [16,17,18]. Within the endosome, acidification triggers the PA prepore to undergo a conformational rearrangement to form a cation-selective, transmembrane pore. EF and LF are translocated across the endosomal membrane through the PA pore. In the cytosol, EF elevates levels of cAMP and causes water retention in the cell [19], and LF cleaves mitogen-activated protein kinase kinases (MAPKKs) [20] (Figure 1).

**Figure 2:**
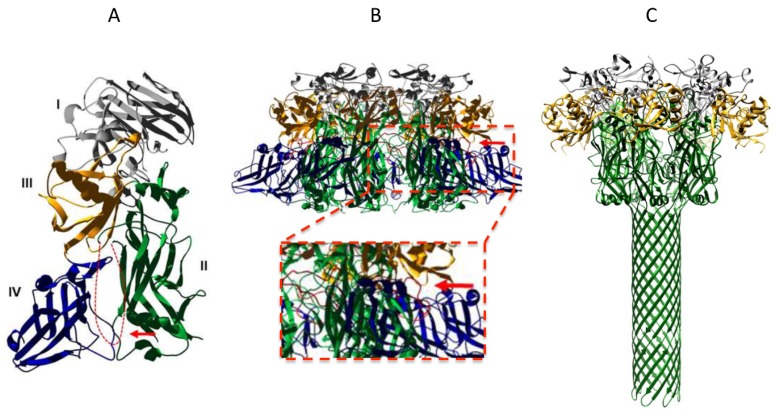
Structures of PA. (**A**) Full-length PA_83_. Four domains are shown in different colors and labeled I–IV. The disordered 2β2–2β3 loop is shown as a red dotted line; (**B**) Upper panel: PA heptameric prepore. Lower panel: a subset of heptameric prepore is zoomed to show the 2β2–2β3 loops that are colored in red and indicated by a red arrow; (**C**) Single particle reconstruction of the PA pore from cryo-EM. The 2β2–2β3 loops convert to a 14-strand transmembrane β-barrel (shown in green). Structural coordinates (1ACC, 1TZO, 3J9C) are downloaded from PDB website, modified and displayed in Swiss-pdb-viewer.

Protein pore formation and membrane translocation is regarded as one of the most challenging and least understood biological processes. In recent years, structural studies as well as biochemical and biophysical characterization of the anthrax toxin in model membranes have achieved significant successes in the understanding of anthrax toxin actions, which has made anthrax toxin an excellent model system for studies of protein membrane translocation. The crystallographic structure of PA_83_ reveals four distinct domains, each of which has a distinct function [21] (Figure 2A). Specifically, domain 2 (D2) forms the transmembrane channel, and domain 4 (D4) is mainly for binding to the receptor [22,23]. The structures of the PA heptameric and octameric prepore have also been solved (Figure 2B) [21,22,24]. The structural and biochemical data support the formation of a 14-strand (heptamer) or 16-strand (octamer) β-barrel during prepore-to-pore conversion. D2 contains a mobile 2β2–2β3 loop (residues 302–322). During the acidic pH-induced conformational rearrangement, seven/eight 2β2–2β3 loops in the PA prepore “peel off” to the base of the structure and form a 14/16-strand transmembrane β-barrel [15,21]. The data from mutagenesis and electrophysiological studies have also proposed a Brownian ratchet model for membrane translocation, in which a Phi (ϕ)-clamp composed of phenylalanine (Phe) 427 residues of PA catalyzes protein translocation in a charge state–dependent mechanism [25,26]. Most excitingly, following a low-resolution EM map of the PA pore [27,28], the long-sought-after atomic structure of the PA pore was recently solved at 2.9-Å resolution by cryo-electron microscopy with direct electron counting (Figure 2C) [29]. The atomic structure of the PA pore shows the catalytic ϕ-clamp and membrane-spanning translocation channel, and supports the Brownian ratchet model.

## 3. Anthrax Toxin Receptor 1 and 2

Anthrax toxin receptors are essential for anthrax toxin action [7,8]. On the cell surface, they serve as a high-affinity binding anchor for the toxin and provide the toxin with a path of entry into the cells. More interestingly, anthrax toxin receptors not only act as a passive binding partner of PA, but also actively function as a molecular clamp or switch by shifting the pH threshold of PA prepore-to-pore conversion to a more acidic pH range, which prevents premature pore formation at neutral pH before the toxin reaches the designated intracellular location [22,30].

**Figure 3:**
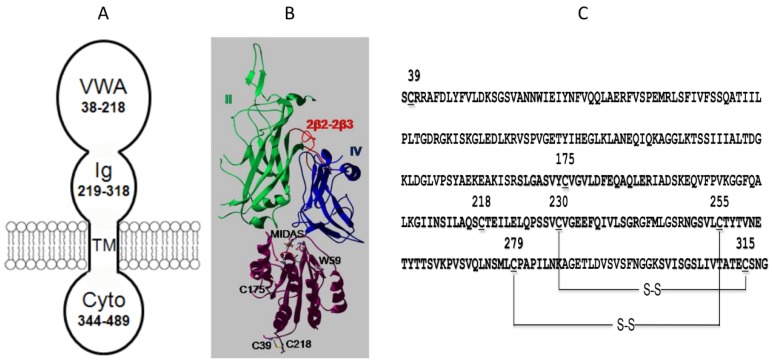
Schematic domains of ANTXR2 and interaction with PA. (**A**) The schematic domains of ANTXR2; (**B**) Interaction of PA and VWA. Domain 2 (green) and domain 4 (blue) of PA and VWA (38–218, purple) are shown. The 2β2–2β3 loop is in red. In the MIDAS motif, Mg^2+^ is coordinated by D50, S52, S54, T118, and D148 (residues shown but not labeled). Disulfide bond C39–C218, unpaired C175, and W59 are shown and labeled. The structure is modified from 1TZN in Swiss-pdb-viewer; (**C**) Sequence of the ANTXR2 ectodomain. The seven conserved Cys residues are underlined. The disulfide bonds C255–C279 and C230–C315 in the Ig domain are labeled. Part of the figure is modified from [37].

Both ANTXR1 and ANTXR2 are type I transmembrane proteins, which are composed of a conserved extracellular ectodomain, a single-pass transmembrane domain and a cytosolic domain (Figure 3A). The ectodomain is the PA-binding domain, while the transmembrane domain and cytosolic domain seem to not be required for anthrax toxin action, since fusion of the ectodomain to a GPI (glycophosphatidylinositol)-linker on the membrane does not affect anthrax toxin action [31]. While ANTXR1 and ANTXR2 share 60% homology in the ectodomain, they show a different binding affinity to PA, with ANTXR2’s binding affinity to PA being at least 1000 folds higher than that of ANTXR1 [32]. Consistently, ANTXR1 and ANTXR2 set different pH thresholds for PA prepore-to-pore conversion. The ANTXR1-bound PA prepore converts to pore at pH ~6 in the early endosome, while the ANTXR2-bound PA prepore converts to pore at pH ~5 in the late endosome [33,34]. By generating ANTXR2-/- and ANTXR1-/- null mice, an important finding was made to reveal the differential roles of the two receptors in anthrax infection *in vivo*. Strikingly, the ANTXR2-/- mice were able to survive a lethal toxin challenge, but the ANTXR1-/- mice were as sensitive to the lethal toxin as wild-type control mice. This study has proved that ANTXR2 is the major receptor for anthrax toxin *in vivo*, and ANTXR1 only plays a minor role [35,36].

## 4. The VWA Domain of ANTXR2

The ectodomain of ANTXR2 is composed of a von Willebrand Factor A domain (VWA, residues 38–218) and an immunoglobulin-like domain (Ig, residues 219–318) (Figure 3A) [37]. The interaction between VWA and PA has been well characterized. VWA binds to PA in 1:1 stoichiometry with a 170 pM affinity in the presence of Mg^2+^ ion [32]. The binding affinity is even increased at low pH, likely through a change in structure that favors a more “bound-like” conformation [38]. The VWA domain shows structural homology with the integrin receptor I domain, which is featured by a Rossmann fold and a conserved metal ion-dependent association site (MIDAS) (Figure 3B). The structure of the PA-VWA complex shows that MIDAS not only binds to the receptor-binding domain D4, but also binds to the pore-forming domain D2 of PA via a Mg^2+^ ion, which provides direct structural evidence that VWA acts as a molecular clamp or switch that controls PA prepore-to-pore conversion (Figure 3B) [22,39]. Recently, numerous studies have uncovered the roles of VWA in PA pore formation with great molecular detail. Using a fluorescence assembly assay, a study shows that VWA drives PA oligomerization and stabilizes the PA heptameric and octameric prepore [15]. Using a histidine hydrogen-deuterium exchange (HDX) method, which monitors the slow rate of HDX of the C_2_ hydrogen of the imidazole group of histidine, studies have shown that VWA increases the stability of PA, and the stabilizing effect reaches up to 70 Å from the receptor-binding interface. This suggests that when PA is anchored at one end, the PA structure is tightened, which is featured by strengthened non-covalent interactions and increased global stability [40,41]. Expectedly, mutations on PA also affect interaction with VWA. While incorporation of 2-fluorohistidine (2-FHis) into PA did not affect pH-dependent pore formation of the soluble PA prepore, when bound to the receptor, the fluorinated PA failed in pore formation and membrane translocation [42]. Moreover, the residue G342 of PA was found to be involved in receptor-specific binding and pore formation. Replacement of G342 with a hydrophobic residue, such as valine, leucine, isoleucine or tryptophan, increased the binding of PA to ANTXR1-expressing cells and decreased the binding of PA to ANTXR2-expressing cells [43].

## 5. The Ig-Like Domain of ANTXR2

**Figure 4:**
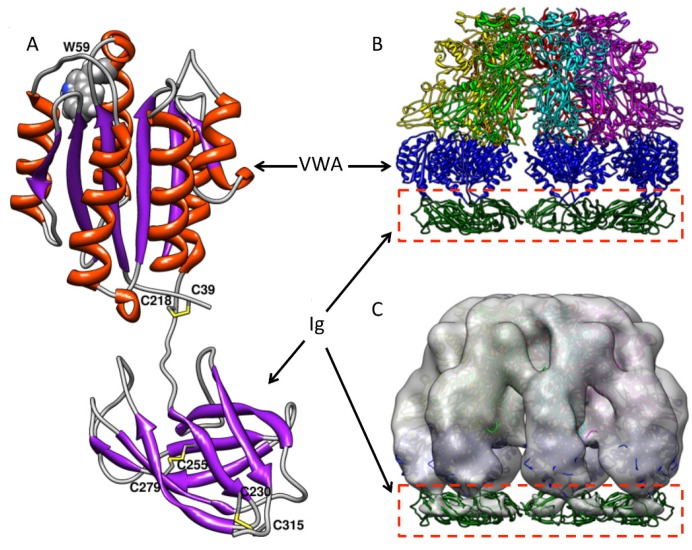
The modeled structure of Ig and the reconstructed EM maps of PA-receptor complex docked with the atomic structure. (**A**) The Ig structure is modeled by homologous modeling and grafted to the crystal structure of VWA with energy minimization. The secondary structure is colored as follows: α-helices, orange; β-sheets, purple; and loops, grey. The disulfide bonds C39–C218, C255–C279 and C230–C315 are shown and colored in yellow; (**B**) PA-receptor heptameric complex, including the modeled Ig domain, was shown as a ribbon structure (side view); (**C**) After 3D reconstruction of negatively stained PA-receptor heptameric complex, the surface-rendered density map was docked with the atomic structure (side view). VWA and Ig are colored in blue and green, respectively. The figure is modified from [45].

Unlike VWA, which has drawn much attention for structural and functional studies, the stalk region underneath VWA had long been ignored. Little was known about its structure and role in anthrax toxin action until it was recently recognized as an immunoglobulin-like domain (Ig), in which the disulfide bonds are required for PA pore formation [37]. There are a total of seven conserved cysteine residues in the ectodomain of ANTXR2, with three on VWA and four on Ig. Expression and purification of the recombinant ANTXR2 ectodomain with both VWA and Ig turned out to be very challenging, which was primarily due to misfolding of the protein in *E. coli* caused by non-specific disulfide cross-linking. The technical obstacle was overcome by fusing the ectodomain with trigger factor (TF), a bacterial foldase, and expression of the fusion protein in *E. coli* Origami B cells. In Origami B cells, both glutathione reductase and thioredoxin reductase are deleted so that the cytosol becomes favorable for disulfide bond formation [44]. Site-directed mutagenesis and mass spectrometry of the recombinant protein demonstrate that the two disulfide bonds in the Ig domain are formed by C255–C279 and C230–C315, respectively (Figure 3C) [45]. While a high-resolution structure of the Ig domain is still not available, computational modeled structures and the low-resolution EM maps have revealed valuable structural details (Figure 4A). Two modeled structures from two different groups show high similarity [45,46]. Moreover, a low-resolution EM map (~14 Å) of the PA-ectodomain heptameric complex was determined by single-particle 3D reconstruction of negatively stained samples (Figure 4B,C). The structural model of the Ig domain generated by homology modeling fits into the additional density underneath the complex. Considering the molecular weight, interestingly, the density of Ig in the EM map was significantly weaker than expected. This suggests that Ig is flexible in solution relative to PA and VWA [45]. This observation is consistent with the structural model, where the long hinge region between VWA and Ig flexible.

**Figure 5:**
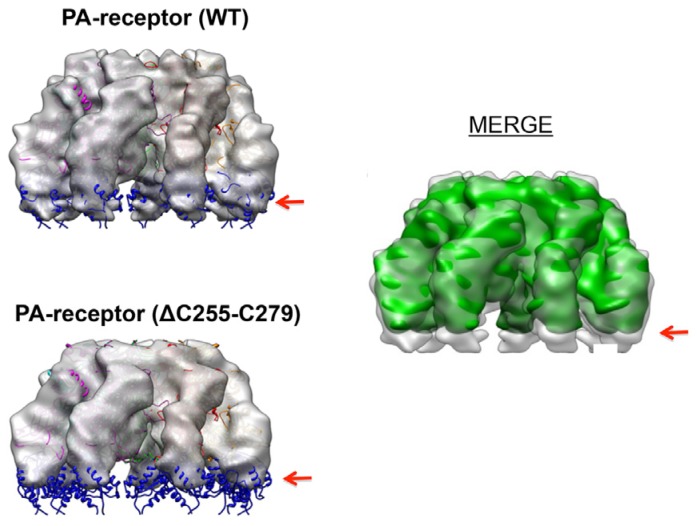
The 3D EM models of PA-receptor (WT) and PA-receptor (ΔC255–C279A) detected the conformational changes on Ig and VWA that are induced by disulfide bond deletion. Upper left: the surface-rendered density map of PA-receptor heptameric complex (side view). Lower left: the surface-rendered density map of PA-receptor (ΔC255–C279A) (side view). The EM maps were docked with the crystal structure of the PA-VWA heptameric complex. The VWA domains are colored in blue. Middle right: the superposed density maps from PA-receptor (WT) (transparent grey) and PA-receptor (ΔC255–C279A) (solid green). Red arrow indicates the position of VWA. The figure is modified from [45].

**Figure 6:**
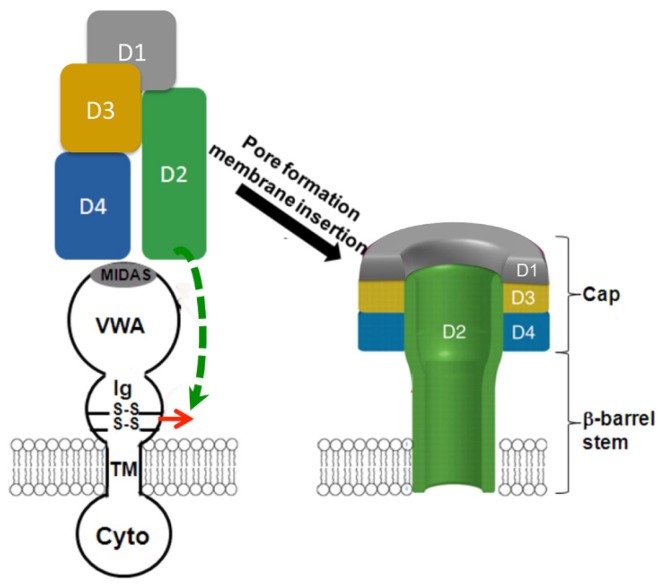
The proposed model by which deletion of the receptor disulfide bond blocks membrane insertion of PA pore. PA binds to the MIDAS motif in VWA. Deletion of the disulfide bond C255–C279 causes significant conformational change on Ig and VWA, which traps the membrane-inserting loop on D2 into the receptor hydrophobic pockets, hence inhibiting PA pore membrane insertion. D1–D4 represent the domains 1–4 of PA.

Site-directed mutagenesis and biochemical characterization have confirmed that the disulfide bond C255–C279, but not C230–C315, is essential for PA pore function [45]. Interestingly, deletion of C255–C279 did not cause defects in PA-receptor binding and PA prepore-to-pore conversion. Instead, the PA membrane-inserting loops were trapped in proteinaceous hydrophobic pockets of the unfolded receptor [45]. Deletion of the disulfide bond not only partly unfolds Ig, but also causes significant conformational changes on the remote site of VWA. The conformational changes were subsequently confirmed by the 3D reconstruction of the single-particle EM maps (Figure 5). The data from fluorescence and structural analysis have concluded that when the disulfide bond C255–C279 is deleted, both Ig and VWA undergo significant conformational changes, which traps the membrane-inserting loops of D2 into the hydrophobic pockets of the unfolded receptor domains (Figure 6) [45]. This study reveals a novel mechanism by which anthrax toxin action may be regulated through manipulating the redox states of the receptor disulfide bonds.

## 6. Does the Receptor Remain Bound to PA after Prepore-to-Pore Conversion?

While it is well established that the receptor affects PA pore formation, it is still not clear whether or not the receptor remains bound to the PA pore and influences the PA pore function after prepore-to-pore conversion. Several independent lines of studies have suggested that receptors are dissociated from the PA pore after conversion. In an earlier study both ANTXR1 and ANTXR2 were co-precipitated with the PA prepore, but not with the PA pore [33]. Later, the data obtained from a study using one-dimensional nuclear magnetic resonance and 2-fluorohistidine labeling also argue for dissociation of the receptor from the PA pore [47]. A recent study used biolayer interferometry and surface plasmon resonance to measure the kinetics of the PA prepore-to-pore transition on the immobilized complexes of the PA prepore with LF_N_. When the PA-LF_N_ complex was bound to VWA, the magnitude of the signal was decreased following acidification, indicating VWA dissociation from the pore [48]. Moreover, the binding of VWA to the PA pore was significantly weaker than the binding to the PA prepore *in vitro* [48]. All of the above-mentioned evidence argues that the receptor dissociates from PA upon prepore-to-pore conversion.

Interestingly, several other independent studies suggest that receptors are not dissociated from PA upon prepore-to-pore conversion. Earlier, the patch clamp was used to measure the ion conductance by the PA pore either on whole cells (with receptors) or on artificial membranes (without receptors). The data showed that the ion conductance of the PA pore was affected by the presence or absence of the receptors. Specifically, the receptor-bound PA pore on the cell membranes has different properties than the PA pore on the artificial membranes in voltage-dependent inactivation and sensitivity to small molecular inhibitors [30]. Later, in immune-precipitation experiments, ANTXR1 and ANTXR2 were labeled with the epitope tags HA and V5, respectively, at their cytosolic domains. Using the antibodies against the epitopes, both PA prepore and pore were found to be co-precipitated with either ANTXR1 or ANTXR2 [17,18]. Moreover, when the D4 domain of PA was expressed and purified as a recombinant protein, it was able to bind VWA at both pH 7 and pH 5 [23]. Most recently, the transfer cross-saturation NMR approach was used to measure the dynamic interaction between PA and VWA during prepore-to-pore conversion. It showed that prior to pore conversion, the contact between VWA and the D2 domain of the PA was weakened, but upon pore conversion, VWA remained bound to the D4 domain of PA. This study indicates that the receptor stabilizes the PA pore upon conversion [49].

In summary, due to the presence of conflicting data, it is difficult to draw a conclusion on whether or not the receptor remains bound to PA after pore formation. The interaction between PA and the receptor, especially upon prepore-to-pore conversion, is a highly dynamic event, which requires more studies to elucidate the mechanism.

## 7. Receptor Decoy and RNAi for Anthrax Toxin Inhibition

Since VWA binds to PA with a high affinity (~170 pM), the soluble VWA has been tested as a receptor decoy to inhibit anthrax toxin cytotoxicity [50,51]. It has been shown that the recombinant VWA not only efficiently neutralized wild-type PA, but also neutralized the altered forms of PA that were modified and became resistant to therapeutic monoclonal antibodies. Several groups fused VWA to the Fc portions of either human IgG1 or IgG2 and generated the recombinant receptor decoys that have a longer circulation half-life *in vivo*, hence increasing antitoxin potencies [52,53]. The VWA-IgG2 protein exhibited a protective effect on the rats that were challenged by the anthrax lethal toxin. The VWA-IgG2 also protected the mice that were infected by the attenuated *B. anthracis* Sterne spores. Importantly, the VWA-IgG1 protein protected rabbits against death by the fully virulent *B. anthracis* Ames spores through an inhalational infection. Recently, several different VWA-based receptor decoy-Ig fusion proteins were engineered and tested both *in vitro* and *in vivo*. When the rats were infected with a lethal dose of anthrax lethal toxin, co-administration of the decoy inhibitors failed to protect the rats from being killed, but the killing was significantly delayed [54].

Most recently, targeted RNA interference (RNAi) technology has been used to inhibit anthrax toxin intoxication through silencing ANTXR1 and/or ANTXR2 in mouse and human macrophages [55]. Similar to pre-treating cells with the specific PA antibody, silencing of ANTXR2 in murine and human macrophages inhibited cytotoxicity and cell death induced by the anthrax lethal toxin. The siRNAs that were designed to target to ANTXR1 also offered significant protection against the lethal toxin. In human kidney cells, silencing of ANTXR2, ANTXR1, or both inhibited the LF-mediated MEK2 cleavage or the EF-catalyzed intracellular cAMP increase.

## 8. Summary

Identification of anthrax toxin receptors is an important milestone in the study of anthrax toxin. Since then, more than a decade of intensive research efforts have produced a great deal of knowledge related to structural and functional aspects of the receptors and have greatly enhanced our understanding of anthrax toxin action, particularly PA pore formation and membrane translocation. Anthrax toxin receptors set an excellent example as the receptors that can actively regulate ligand-binding, endocytosis and pH-dependent conformational changes. More discoveries are expected in the future to reveal the physiological roles of the receptors.
